# Parasitomimetics: Can We Utilize Parasite-Derived Immunomodulatory Molecules for Interventions to Immunological Disorders?

**DOI:** 10.3389/fimmu.2022.824695

**Published:** 2022-03-21

**Authors:** Kazuki Nagai, Yasuyuki Goto

**Affiliations:** Laboratory of Molecular Immunology, Graduate School of Agricultural and Life Sciences, The University of Tokyo, Tokyo, Japan

**Keywords:** parasites, hygiene hypothesis, immunological disorders, immunomodulatory molecules, therapeutic interventions

## Abstract

Because our immune system has ability to expel microorganisms invading our body, parasites need evolution to maintain their symbiosis with the hosts. One such strategy of the parasites is to manipulate host immunity by producing immunomodulatory molecules and the ability of parasites to regulate host immunity has long been a target of research. Parasites can not only manipulate host immune response specific to them, but also influence the host’s entire immune system. Such ability of the parasites may sometimes bring benefit to the hosts as many studies have indicated the “hygiene hypothesis” that a decreased opportunity of parasitic infections is associated with an increased incidence of allergy and autoimmune diseases. In other words, elucidating the mechanisms of parasites to regulate host immunity could be applied not only to resolution of parasitic infections but also to treatment of non-parasitic immunological disorders. In this review, we show how much progress has been made in the research on immunomodulation of host immunity by parasites. Here, we define the word ‘parasitomimetics’ as emulation of parasites’ immunomodulatory systems to solve immunological problems in humans and discuss potential applications of parasite-derived molecules to other diseases.

## Introduction

Parasitism, one form of symbiosis, is a relationship between species where one species receives benefit from the host while causing harm to the host. Infection with parasites causes parasitic diseases which have been major health threats to humans for a long time. There are three main classes of parasites that can cause disease in humans: protozoa, helminths, and ectoparasites. Even in this modern era, more than 1.5 billion people, or 24% of the world’s population, are infected with soil-transmitted helminth infections worldwide (1).

Majority of parasites cause chronic infections, i.e., sustained survival in their hosts. This means that the hosts have enough time to recognize the invading foreign bodies and establish immunity to expel the parasites. The host immune response can be classified into innate and acquired immunity, and the host defends itself against pathogens with a variety of strategies. In contrast, parasites, which require chronic infection of the host, have often been able to escape the host immunity by inducing immune modulation in the host. In other words, as the host evolves its own immune mechanisms, the parasite evolves its own mechanisms to evade immunity. The ability of these parasites to regulate host immunity has long been a target of research.

Helminths are the representative parasites that regulate host immunity with diverse strategies. One of the strategies is to mimic host molecules which are involved in immunosuppression. An example is TGF-β which induces regulatory T cells and inhibits effector functions in NK cells and macrophages to suppress host immunity (2), and it is known that helminths have a homologue of a mammalian TGF-β; a TGF-β homologue was discovered in *Brugia malayi* and named Bm-TGH-2 (3). Other helminth-derived molecules such as FhTLM from *Fasciola hepatica* and HpTGM from *Heligmosomoides polygyrus* have been identified and are involved in T cell suppression through binding to TGF-β receptors on T cells and its signaling (4, 5). Furthermore, FhTLM binds not only to TGF-β receptors on T cells but also to TGF-β receptors on macrophages and induces IL-10 secretion by macrophages (4). Interestingly, although HpTGM is a protein with cytokine-like functions, it has no homology to mammalian TGF-β or other members of the TGF-β family (5). Thus, parasites have molecules that functionally play similar roles to host molecules, even though they are not structurally conserved.

Another example of parasite secretory molecules mimicking host molecules is macrophage migration inhibitory factor (MIF). MIF is a cytokine that causes inflammation and migration of immune cells, and is mainly expressed by pituitary corticotropic cells, monocytes/macrophages, and T cells (6, 7). MIF causes allergic reactions by inducing pro-inflammatory cytokines such as TNF-α, IL-1β, and IL-8 (8, 9). Many homologs of MIF have been discovered, such as Bm-MIF from *B. malayi* (10), TsMIF from *Trichinella spiralis* (11), AceMIF from *Ancylostoma ceylanicum* (12), and AsMIF from *Anisakis simplex* (13). However, functions of the parasite-derived MIFs are respectively diverse. Bm-MIFs, like host MIFs, induce IL-8 and TNF-α, leading to inflammation (10). Bm-MIF also induces host MIF, enhancing MIF-mediated immune responses (10). On the other hand, TsMIF and AceMIF are known to bind to host MIF receptors, but their detailed functions are not known (11, 12). Furthermore, AsMIF not only regulates macrophages by reducing Th2-related cytokines, but also induces regulatory T cells and immunosuppression by production of IL-10 and TGF-β (13). Thus, even if parasite molecules have sequence homology to host molecules, their functions are not necessarily conserved.

Parasite molecules that resemble host molecules are not limited to cytokines. Cystatin acts as a cysteine protease inhibitor *in vivo*. In the immune system, cystatins inhibit cysteine proteases involved in antigen processing such as lysosomal cathepsins and asparaginyl endopeptidase (AEP) (14). Some homologs of cystatin have been identified in parasites. Bm-CPI-2 and Al-CPI from *B. malayi* and *Ascaris lumbricoides*, respectively, inhibit host cysteine proteases and suppress antigen presentation (15, 16). Hp-CPI from *H. polygyrus* inhibits antigen and MHC class II molecule processing and induces immunosuppression (17). LsCystatin from *Litomosoides sigmodontis* reduces nitric oxide production (18) and onchocystatin from *Onchocerca volvulus* induces IL-10 production and suppresses antigen-stimulated cell proliferation (19).

As mentioned earlier, it has also been reported that parasite molecules structurally dissimilar to host molecules may act directly on the host immune system to induce immunomodulation. Omega-1 is a glycoprotein which is secreted from *Schistosoma mansoni* eggs. Omega-1 acts as T2 ribonuclease (RNase) and prevents protein synthesis by decomposing of ribosome RNA or messenger RNA (20). This RNase activity plays a role in conditioning DCs to prime Th2 responses (21, 22). CP1412, a close homolog to omega-1 from *Schistosoma japonicum*, also induces Th2 cell polarization through the RNase activity (23).

Although it has been reported that *S*. *mansoni* soluble egg antigen prevents type 1 diabetes in non-obese diabetic (NOD) mice (24), the identification of omega-1 leads to possibility to utilize parasite-derived molecules as therapeutic agents of type 1 diabetes. Injection of recombinant omega-1 into NOD mice induces expression of IL-4 and Foxp3 (25). Moreover, injection of the molecule into obese mice improves insulin sensitivity by IL-33 release from white adipose tissue (26). These results indicate that parasite molecules, even structurally dissimilar to host molecules, can be useful for therapy of immune-mediated diseases.

Together, parasites have potential to manipulate the host immunity. Here, we would like to shift the focus to protozoa. One of the characteristics of protozoan parasites is their intracellular parasitism to various types of host cells, and especially those living in phagocytic cells may require the highest level of immune escape by immunomodulation. Here, we focus on protozoa that parasitize phagocytic cells (macrophages, DCs), *Toxoplasma gondii*, *Leishmania* spp. and *Trypanosoma cruzi*, and review immunomodulatory molecules and their mechanisms of action and potential of the parasite-derived immunomodulators as drugs for immunological disorders.

## Immunomodulatory Molecules and Their Mechanisms of Action

### Toxoplasma gondii

*Toxoplasma gondii* is a eukaryotic parasite that can only grow in host cells. *T*. *gondii* is one of the most widespread apicomplexans and is a common parasite of animals and humans. It is well known to cause serious opportunistic infections. Tachyzoites of *T*. *gondii* can infect any cell types but erythrocytes and can evade host immunity of macrophages or DCs (27, 28).

Rhoptry proteins of *T*. *gondii* are secreted from rhoptry and are associated with invasion of the parasites into host cells. ROP16 is one of the rhoptry proteins which was originally identified as a serine-threonine kinase (29), and then was shown to directly phosphorylates host STAT3/6, suppresses innate immune cytokine secretion (29, 30), and to induce macrophages to become M2 macrophages (30, 31). Other rhoptry proteins that directly act on host immunity include ROP18, which inhibits IRG-mediated clearance of macrophages (32). ROP18 phosphorylates p65 and inhibits NF-κB, resulting in suppression of inflammatory cytokines (32, 33).

Dense granule proteins secreted by *T*. *gondii* may manipulate host inflammatory responses. GRA18 has been identified as the parasite’s anti-inflammatory molecule; once released in the host cell cytoplasm, it forms complexes with regulatory elements of the β-catenin destruction complex and prevents β-catenin from being destroyed (34). Within macrophages, GRA18 induces expression of a specific set of genes commonly associated with anti-inflammatory responses, including genes encoding the chemokines CCL17 and CCL22 (34). *T. gondii* inhibitor of STAT1 transcriptional activity (TgIST) binds to activated STAT1 dimers in the nucleus of IFN-γ-treated cells and represses the IFN-γ–mediated STAT1-dependent proinflammatory expression (35).

### *Leishmania* spp.

Leishmaniasis is a disease caused by protozoan parasites of the genus *Leishmania*. The parasites are transmitted to the mammalian host by sand fly. There are three main forms of leishmaniasis, cutaneous leishmaniasis, mucocutaneous leishmaniasis and visceral leishmaniasis. *Leishmania* proliferates within macrophages in their mammalian hosts. Because macrophages have ability to kill internalized pathogens, it is likely that *Leishmania* has acquired a sophisticated system for evading the host immunity by directly controlling the immune system.

*Leishmania* GP63 is a metalloprotease that causes cleavage of various peptides. It has already been reported to cleave several host immune-related proteins such as MARCKS-related protein (36), mTOR (37), NF-κB p65 (38), PTP and SHP-1 (39). Cleavage of PTP results in stimulation of phosphatase activity, which leads to rapid downregulation of Janus kinase and mitogen-activated protein kinase signaling (39). GP63 also cleaves mTOR and dephosphorylates 4E-BP1, resulting in transcriptional repression and macrophage suppression (37). SHP-1-mediated suppression of macrophages is dependent not only on cleavage by GP63, and another leishmanial protein elongation factor-1alpha (EF-1α) binds to and activates SHP-1, which in turn inhibits macrophage activation (40, 41). This ability of *Leishmania* to inhibit macrophage effector functions may result from direct interference by *Leishmania* molecules such as GP63 and EF-1α with macrophage signal transduction pathways.

TGF-β prevents macrophage activation and exacerbates *Leishmania* infections (42). Some species of *Leishmania* have been known to trigger the production of biologically active TGF-β by macrophages (42, 43). The latent TGF-β1 is activated by treatment with proteases such as plasmin and cathepsin D. It has been reported that *Leishmania* parasites contain high levels of cysteine proteases, belonging to cathepsin L and cathepsin B families (44). Cathepsin B-like cysteine protease from *Leishmania donovani* is able to cleave latent TGF-β1 into an active form releasing latency-associated protein (45).

Arginase encoded in *Leishmania* may manipulate the polarity of host macrophages. Macrophages harbor two competing pathways for arginine metabolism initiated by the enzymes inducible NO synthase (iNOS) and arginase, respectively. Mammalian arginase-I hydrolyzes arginine to urea and ornithine, and macrophages dominated by the arginase pathway, in other words alternatively activated macrophages, have suppressed iNOS production resulting in defects in nitric oxide-mediated parasite killing. Parasite-derived arginase seems to cause local depletion of the iNOS substrate arginine and to enhances parasite survival (46, 47).

Similar to helminths, *Leishmania* and *Toxoplasma* also encode MIF homologs (48). *Leishmania* MIF upregulates inflammatory and innate immune signaling in infected macrophages including inflammatory genes such as cxcl1, tlr2, and tnf (49). *Toxoplasma* MIF induces IL-8 production by human peripheral blood mononuclear cells and activates the ERK/MAPK pathway in mouse bone marrow-derived macrophages (50).

### Trypanosoma cruzi

Chagas disease is a chronic disease caused by infection with *Trypanosoma cruzi*. The parasites are transmitted to the mammalian host by reduviid bug. Some people with chronic *T*. *cruzi* infection eventually develop clinical disease including predominantly cardiac dysfunction.

One of the characteristics of *T*. *cruzi* infection is polyclonal activation of B cells (51). The polyclonal B cell activation could restrict optimal development of antigen-specific lymphocytes involved in protective responses to the infection by increasing competition for activation and survival signals in the lymphoid tissues (52). *T*. *cruzi* proline racemase was identified as a B cell mitogen which activates B cells polyclonally (53). Moreover, its racemase active site is necessary for mitogenic activity and it was a new finding that the eukaryotic amino acid racemase has potential to activate B cells (53). *T*. *cruzi* trans-sialidase (TcTS) is also known as a B cell mitogen that causes T cell-independent B cell activation and induces non-specific immunoglobulin secretion (54). TcTS also promotes IL-17 production by activated B cells (55). *T. cruzi* P21 binds to CXCR4 and activates actin polymerization and macrophage phagocytosis through PI3-kinase signaling pathway (56, 57). This favors phagocytosis of the parasites by macrophages. Furthermore, P21 induces recruitment of leukocytes to the site of inflammation and up-regulates expression of IL-4 inducing Th2 immune response (58).

*T. cruzi* trypomastigotes are complement-resistant (59, 60). *T. cruzi* calreticulin (TcCRT) inhibits both the classical and lectin complement pathways (61, 62). It binds and inactivates the first complement component C1 and mannose-binding lectin (62). TcCRT prevents C1 formation by interfering binding of C1r and C1s to C1q (63). TcCRT also binds to the L-ficolin collagenous portion and prevents the human complement lectin pathway activation (62). Moreover, in mammals, the binding of C1q to calreticulin is a so-called “eat-me signal” that can recruit macrophages and initiate the apoptotic process (64). TcCRT promotes infectivity of *T. cruzi* and is essential for the parasites to invade host macrophages (65). Together, TcCRT, which mimics host calreticulin, may be important for survival of *T. cruzi* by ensuring efficient entry to macrophages without inducing their parasite-killing activities.

The main cysteine peptidase from *T. cruzi* is cruzipain, a papain-like endopeptidase expressed in all life cycle stages of the parasite (66). Cruzipain is able to activate latent TGF-β *in vitro* (67). Activation of TGF-β is required for parasite entry into the mammalian cells and play an important role in cardiomyocyte invasion by *T. cruzi* (68). A moderate oxidative environment is advantageous for *T. cruzi* proliferation (69) and Chagas disease is known to cause an increase in reactive oxygen species (ROS) in infected tissues and cells (70). Intracellular *T. cruzi* forms release in the host cytosol its major cyclophilin of 19 kDa (TcCyp19) (71). TcCyp19, causes the increase in ROS required for parasite growth in the mammalian host (71).

## Potential of Parasite-Derived Immunomodulators as Drugs for Immunological Disorders

Though parasites’ skills to modulate host immunity are beneficial for their own survival, situations where such immunomodulatory molecules are highly appraised may go beyond infections. The “hygiene hypothesis” states that as exposure to pathogens decreases due to improved sanitation, the risk of allergic and autoimmune diseases increases. This hypothesis is based on the fact that the large increase in the frequency of allergic and autoimmune diseases observed in developed countries over the past 40 years is negatively correlated with an overall decrease in the frequency of infectious diseases (72). In experimental models, the occurrence of autoimmune disease is prevented by infection with distinct pathogens, such as bacteria, virus and parasite (72).

There have already been attempts to treat immune-mediated diseases by artificially infecting them with helminths. Because *Trichuris suis* can be obtained from experimentally infected pigs, the parasite has been used in immunotherapy research to artificially infect people. There are some clinical reports that patients who ingested *T. suis* had a reduction in several immune disorders such as Crohn’s disease (73) and ulcerative colitis (74). However, some clinical trials have shown no therapeutic effect (75, 76) and in a large study using meta-analysis, *T. suis* showed no apparent benefit for inflammatory bowel disease patients (77). Besides, therapeutic benefit by parasites may be limited to the infection sites but not systemic. A clinical trial of artificial infection with *T. suis*, a parasite of the intestine, did not provide relief from allergic rhinitis (78). Furthermore, infection with live parasites for therapeutic use may not be practical, and can sometimes cause other unintended consequences. There is also a risk of inadvertently transmitting pathogenic parasites. For this reason, it is imperative to identify immunomodulatory molecules and apply them to treatment of immune-mediated diseases rather than using live parasites. In order to ensure safety, it is also necessary to elucidate the detailed mechanism of how the molecule regulates immunity.

Using helminth molecules that regulate host immunity could have the potential to alleviate the symptoms of immune disorders. As mentioned above, helminth cystatins exhibit host immunosuppression through a variety of mechanisms. Recombinant proteins of *B. malayi* and *Clonorchis sinensis* cystatin showed anti-inflammatory activity and significantly reduced symptom of dextran sulfate sodium (DSS)-induced colitis in mice (79, 80). The recombinant cystatins down-regulated expressions of IFN-γ and TNF-α and up-regulated IL-10 expression in the DSS-induced colitis mice (79, 80). Administration of recombinant *S. japonicum* cystatin also reduced inflammatory parameters and ameliorated the severity of the trinitrobenzene sulfonic acid (TNBS)-induced colitis (81). HpTGM from *H. polygyrus* is a TGF-β mimicry and suppresses intestinal inflammatory response (5, 82). In Smyth’s study (2020), active HpTGM was recombinantly expressed by *Chlamydomonas reinhardtii* in order to be safely consumed orally in mice and humans. Oral administration of recombinant HpTGM regulated immune cells and ameliorated weight loss, lymphadenopathy, and disease symptoms in a mouse model of DSS-induced colitis (82).

This section explains the status of research on how the protozoan parasites introduced in the previous chapter can be applied to the treatment of immune-mediated diseases. *T. gondii* can be divided into distinct clonal lines: types I, II and III and each strain has a different virulence (83–85). ROP16 of *T. gondii* type I and III (ROP16 I/III) induces M2 macrophages to ameliorate inflammatory bowel disease (86). This study was based on the fact that ROP16 I/III induce deflection of RAW264.7 to M2 macrophages and suppress M1-related inflammatory responses (86, 87). In fact, Caco-2 intestinal epithelial cells co-cultured with M1 macrophages underwent apoptosis, but Caco-2 cells co-cultured with ROP16-transfected macrophages induced to M2 showed suppressed apoptosis (86).

When *Leishmania* is taken up by macrophages, LPS-induced proinflammatory cytokines such as IL-12, IL-17, and IL-6 are specifically suppressed by the parasite (88). *L. donovani* infection of monocytes causes suppression of TLR2 and TLR4-stimulated IL-12, with an increase in IL-10 production (89, 90). Because chronic secretion of inflammatory cytokines is one of the causes of immune-mediated diseases such as atherosclerosis (91), Crohn’s disease (92), and rheumatoid arthritis (93), identification of the leishmanial immunosuppressive factors (components) will lead to the development of therapeutic agents for the immune-mediated diseases in the future. *L. infantum* infection also induces down-regulation of NLRP3 inflammasome activation in Aβ_42_-stimulated cells (94). Activation of the NLRP3 inflammasome is a major play in the neuroinflammation that accompanies Alzheimer’s disease (95) and the down-regulation could prevent disease development.

The potential of parasite-derived molecules is not only to suppress host immunity and alleviate immune diseases. They can also be used to manipulate host immune cells and to prevent other pathogen infection. TcTS may have a potential to remove sialic acid from cellular membranes, and TcTS treatment decreases mycoplasma infection through preventing of adhesion to the glycoproteins which require sialic acid (96). This study was based on the observation that chagasic patients was are less likely to be infected with mycoplasma (96).

## Conclusions

With a recent increase in the prevalence of immune disorders, such as allergic disorders (97, 98), rheumatoid arthritis (99), ulcerative colitis and Crohn’s disease (100, 101), there is an increasing demand on therapeutic interventions for such diseases. Since many of currently available drugs are derived from natural resources, it is also reasonable to seek clues for novel immunomodulatory interventions in parasites, the professional immunomodulatory organisms. Biomimetics is an emulation of models and systems living organisms have and their applications to solve various issues we face. We here propose a new term ‘parasitomimetics’, which is made up with ‘parasite’ and ‘biomimetics’, to represent research activities focusing on parasites’ unique skills and their utilization for tackling medical issues including immune disorders. Parasites have acquired amazing abilities in the process of evolution, e.g., cell adhesion, cell invasion, escape from host cells, and morphological changes/metamorphosis. Among them, parasites’ abilities to regulate host immunity may be one of the most practical for medical applications. However, parasites’ harmful effects to the hosts should be seriously considered when applying them to other diseases. Unlike the usage of live parasites, an approach to mimic parasites’ immunomodulatory skills by identifying the responsible molecules and synthesizing them for administration will lead to more controlled product development/standardization and minimize a risk of adverse events. On the other hand, immunomodulatory effects of parasites are often achieved by more than one molecules and active components can be missed during the identification process if we conceive that the immunomodulatory effect is derived from a single molecule. Besides, immunomodulatory molecules of parasites are not limited to proteins, which are covered in this review. For example, trehalose was identified as a molecule derived from *H. polygyrus* that induce CD8^+^ Treg cells leading to prevention of autoimmune-mediated diabetes (102). Some cautions and novel systematic methods in identifying divergent and/or multi-component immunomodulators may increase the success rate of parasitomimetics.

Like helminths, there is also progress in identifying molecules from protozoa parasites that manipulate host immunity and elucidating their mechanisms. On the other hand, attempts to use them for treatment of immune-mediated diseases are lagging when compared with helminths. Immunomodulatory abilities of the protozoan parasites that have evolved to parasitize within immune cells should be unique and divergent from those of helminths and may be applied to resolving human immune-mediated diseases where helminth-derived molecules cannot cover.

## Author Contributions

KN and YG contributed to conception. KN wrote the first draft of the manuscript. YG reviewed the draft. KN and YG contributed to manuscript revision. All the authors read and approved the submitted version.

## Funding

This work was supported by KAKENHI (18H02649, 20K21516, 21H02722 to YG) from Japan Society for the Promotion of Science.

## Conflict of Interest

The authors declare that the research was conducted in the absence of any commercial or financial relationships that could be construed as a potential conflict of interest.

## Publisher’s Note

All claims expressed in this article are solely those of the authors and do not necessarily represent those of their affiliated organizations, or those of the publisher, the editors and the reviewers. Any product that may be evaluated in this article, or claim that may be made by its manufacturer, is not guaranteed or endorsed by the publisher.

## References

[1] World Health Organization. Fact Sheet. Available at: https://www.who.int/news-room/fact-sheets/detail/soil-transmitted-helminth-infections (Accessed November 23, 2021).

[2] Chen W Ten Dijke P. Immunoregulation by Members of the Tgfβ Superfamily. Nat Rev Immunol (2016) 16:723–40. doi: 10.1038/nri.2016.112 27885276

[3] Gomez-Escobar N Gregory WF Maizels RM. Identification of Tgh-2, a Filarial Nematode Homolog of Caenorhabditis Elegans Daf-7 and Human Transforming Growth Factor β, Expressed in Microfilarial and Adult Stages of Brugia Malayi. Infect Immun (2000) 68:6402–10. doi: 10.1128/IAI.68.11.6402-6410.2000 PMC97726 11035752

[4] Sulaiman AA Zolnierczyk K Japa O Owen JP Maddison BC Emes RD. Flynn RJ. A Trematode Parasite Derived Growth Factor Binds and Exerts Influences on Host Immune Functions *via* Host Cytokine Receptor Complexes. PloS Pathog (2016) 12:1–22. doi: 10.1371/journal.ppat.1005991 PMC5091765 27806135

[5] Johnston CJC Smyth DJ Kodali RB White MPJ Harcus Y Filbey KJ. A Structurally Distinct TGF-β Mimic From an Intestinal Helminth Parasite Potently Induces Regulatory T Cells. Nat Commun (2017) 8:1741. doi: 10.1038/s41467-017-01886-6 29170498 PMC5701006

[6] Alourfi Z Ray DW Donn R. Macrophage Migration Inhibitory Factor (MIF). Cytokine Gene Polymorphisms Multifactorial Cond (2002) 30:191–205. doi: 10.1201/9781420005325.ch14

[7] Bernhagen J Krohn R Lue H Gregory JL Zernecke A Koenen RR. MIF is a Noncognate Ligand of CXC Chemokine Receptors in Inflammatory and Atherogenic Cell Recruitment. Nat Med (2007) 13:587–96. doi: 10.1038/nm1567 17435771

[8] Calandra T Bernhagen J. R a Mitchell RB. The Macrophage is an Important and Previously Unrecognized Source of Macrophage Migration Inhibitory Factor. J Exp Med Rockefeller Univ Press (1994) 179:1895–902. doi: 10.1084/jem.179.6.1895 PMC2191507 8195715

[9] Calandra T Bernhagen J Metz CN Spiegel LA Bacher M Donnelly T. MIF as a Glucocorticoid-Induced Modulator of Cytokine Production. Nature (1995) 377:68–71. doi: 10.1038/377068a0 7659164

[10] Zang X Taylor P Wang JM Meyer DJ Scott AL Walkinshaw MD. Homologues of Human Macrophage Migration Inhibitory Factor From a Parasitic Nematode: Gene Cloning, Protein Activity, and Crystal Structure. J Biol Chem (2002) 277:44261–7. doi: 10.1074/jbc.M204655200 12221083

[11] Tan THP Edgerton SAV Kumari R McAlister MSB Rowe SM Nagl S. Macrophage Migration Inhibitory Factor of the Parasitic Nematode Trichinella Spiralis. Biochem J (2001) 357:373–83. doi: 10.1042/0264-6021:3570373 PMC1221963 11439086

[12] Cho Y Jones BF Vermeire JJ Leng L DiFedele L Harrison LM. Structural and Functional Characterization of a Secreted Hookworm Macrophage Migration Inhibitory Factor (MIF) That Interacts With the Human MIF Receptor CD74. J Biol Chem (2007) 282:23447–56. doi: 10.1074/jbc.M702950200 PMC3707627 17567581

[13] Park SK Cho MK Park H-K Lee KH Lee SJ Choi SH. Macrophage Migration Inhibitory Factor Homologs of Anisakis Simplex Suppress Th2 Response in Allergic Airway Inflammation Model *via* CD4 + CD25 + Foxp3 + T Cell Recruitment. J Immunol (2009) 182:6907–14. doi: 10.4049/jimmunol.0803533 19454687

[14] Pierre P Mellman I. Developmental Regulation of Invariant Chain Proteolysis Controls MHC Class II Trafficking in Mouse Dendritic Cells. Cell (1998) 93:1135–45. doi: 10.1016/S0092-8674(00)81458-0 9657147

[15] Manoury B Gregory WF Maizels RM Watts C. Bm-CPI-2, a Cystatin Homolog Secreted by the Filarial Parasite Brugia Malayi, Inhibits Class II MHC-Restricted Antigen Processing. Curr Biol (2001) 11:447–51. doi: 10.1016/S0960-9822(01)00118-X 11301256

[16] Coronado S Barrios L Zakzuk J Regino R Ahumada V Franco L. A Recombinant Cystatin From Ascaris Lumbricoides Attenuates Inflammation of DSS-Induced Colitis. Parasite Immunol (2017) 39:1–10. doi: 10.1111/pim.12425 28295446

[17] Sun Y Liu G Li Z Chen Y Liu Y Liu B. Modulation of Dendritic Cell Function and Immune Response by Cysteine Protease Inhibitor From Murine Nematode Parasite Heligmosomoides Polygyrus. Immunology (2013) 138:370–81. doi: 10.1111/imm.12049 PMC3719947 23240853

[18] Pfaff AW Schulz-Key H Soboslay PT Taylor DW MacLennan K Hoffmann WH. Litomosoides Sigmodontis Cystatin Acts as an Immunomodulator During Experimental Filariasis. Int J Parasitol (2002) 32:171–8. doi: 10.1016/S0020-7519(01)00350-2 11812494

[19] Schönemeyer A Lucius R Sonnenburg B Brattig N Sabat R Schilling K. Modulation of Human T Cell Responses and Macrophage Functions by Onchocystatin, a Secreted Protein of the Filarial Nematode Onchocerca Volvulus. J Immunol (2001) 167:3207–15. doi: 10.4049/jimmunol.167.6.3207 11544307

[20] Everts B Hussaarts L Driessen NN Meevissen MHJ Schramm G van der Ham AJ. Schistosome-Derived Omega-1 Drives Th2 Polarization by Suppressing Protein Synthesis Following Internalization by the Mannose Receptor. J Exp Med (2012) 209:1753–67. doi: 10.1084/jem.20111381 PMC3457738 22966004

[21] Everts B Perona-Wright G Smits HH Hokke CH van der Ham AJ Fitzsimmons CM. Omega-1, a Glycoprotein Secreted by Schistosoma Mansoni Eggs, Drives Th2 Responses. J Exp Med (2009) 206:1673–80. doi: 10.1084/jem.20082460 PMC2722183 19635864

[22] Steinfelder S Andersen JF Cannons JL Feng CG Joshi M Dwyer D. The Major Component in Schistosome Eggs Responsible for Conditioning Dendritic Cells for Th2 Polarization is a T2 Ribonuclease (Omega-1). J Exp Med (2009) 206:1681–90. doi: 10.1084/jem.20082462 PMC2722182 19635859

[23] Ke XD Shen S Song LJ Yu CX Kikuchi M Hirayama K. Characterization of Schistosoma Japonicum CP1412 Protein as a Novel Member of the Ribonuclease T2 Molecule Family With Immune Regulatory Function. Parasites Vectors (2017) 10:1–19. doi: 10.1186/s13071-016-1962-y 28212670 PMC5316207

[24] Zaccone P Burton O Miller N Jones FM Dunne DW Cooke A. Schistosoma Mansoni Egg Antigens Induce Treg That Participate in Diabetes Prevention in NOD Mice. Eur J Immunol (2009) 39:1098–107. doi: 10.1002/eji.200838871 19291704

[25] Zaccone P Burton OT Gibbs SE Miller N Jones FM Schramm G. The s. Mansoni Glycoprotein ω-1 Induces Foxp3 Expression in NOD Mouse CD4 + T Cells. Eur J Immunol (2011) 41:2709–18. doi: 10.1002/eji.201141429 21710488

[26] Hams E Bermingham R Wurlod FA Hogan AE O’Shea D Preston RJ. The Helminth T2 Rnase ω1 Promotes Metabolic Homeostasis in an IL-33- and Group 2 Innate Lymphoid Cell-Dependent Mechanism. FASEB J (2016) 30:824–35. doi: 10.1096/fj.15-277822 PMC4973506 26490658

[27] Dubremetz JF. Host Cell Invasion by Toxoplasma Gondii. Trends Microbiol (1998) 6:27–30. doi: 10.1016/S0966-842X(97)01165-7 9481821

[28] Sacks D Sher A. Evasion of Innate Immunity by Parasitic Protozoa. Nat Immunol (2002) 3:1041–7. doi: 10.1017/S0031182005008127 12407413

[29] Yamamoto M Standley DM Takashima S Saiga H Okuyama M Kayama H. A Single Polymorphic Amino Acid on Toxoplasma Gondii Kinase ROP16 Determines the Direct and Strain-Specific Activation of Stat3. J Exp Med (2009) 206:2747–60. doi: 10.1084/jem.20091703 PMC2806617 19901082

[30] Butcher BA Fox BA Rommereim LM Kim SG Maurer KJ Yarovinsky F. Toxoplasma Gondii Rhoptry Kinase Rop16 Activates Stat3 and Stat6 Resulting in Cytokine Inhibition and Arginase-1-Dependent Growth Control. PloS Pathog (2011) 7:e1002236. doi: 10.1371/journal.ppat.1002236 21931552 PMC3169547

[31] Chen L Christian DA Kochanowsky JA Phan AT Clark JT Wang S. The Toxoplasma Gondii Virulence Factor ROP16 Acts in Cis and Trans, and Suppresses T Cell Responses. J Exp Med (2020) 217:e20181757. doi: 10.1084/jem.20181757 31961916 PMC7062521

[32] Behnke MS Fentress SJ Mashayekhi M Li LX Taylor GA Sibley LD. The Polymorphic Pseudokinase ROP5 Controls Virulence in Toxoplasma Gondii by Regulating the Active Kinase ROP18. PloS Pathog (2012) 8:e1002992. doi: 10.1371/journal.ppat.1002992 23144612 PMC3493473

[33] Du J An R Chen L Shen Y Chen Y Cheng L. Toxoplasma Gondii Virulence Factor Rop18 Inhibits the Host Nf-Kb Pathway by Promoting P65 Degradation. J Biol Chem (2014) 289:12578–92. doi: 10.1074/jbc.M113.544718 PMC4007449 24648522

[34] He H Brenier-Pinchart MP Braun L Kraut A Touquet B Couté Y. Characterization of a Toxoplasma Effector Uncovers an Alternative GSK3/β-Catenin-Regulatory Pathway of Inflammation. Elife (2018) 7:1–28. doi: 10.7554/eLife.39887 PMC6214654 30320549

[35] Gay G Braun L Brenier-Pinchart MP Vollaire J Josserand V Bertini RL. Toxoplasma Gondii Tgist Co-Opts Host Chromatin Repressors Dampening STAT1-Dependent Gene Regulation and IFN-γ-Mediated Host Defenses. J Exp Med (2016) 213:1779–98. doi: 10.1084/jem.20160340 PMC4995087 27503074

[36] Corradin S Ransijn A Corradin G Roggero MA Schmitz AAP Schneider P. MARCKS-Related Protein (MRP) is a Substrate for the Leishmania Major Surface Protease Leishmanolysin (Gp63). J Biol Chem (1999) 274:25411–8. doi: 10.1074/jbc.274.36.25411 10464270

[37] Jaramillo M Gomez MA Larsson O Shio MT Topisirovic I Contreras I. Leishmania Repression of Host Translation Through Mtor Cleavage is Required for Parasite Survival and Infection. Cell Host Microbe (2011) 9:331–41. doi: 10.1016/j.chom.2011.03.008 21501832

[38] Gregory DJ Godbout M Contreras I Forget G Olivier M. A Novel Form of NF-κb is Induced by Leishmania Infection: Involvement in Macrophage Gene Expression. Eur J Immunol (2008) 38:1071–81. doi: 10.1002/eji.200737586 18383035

[39] Gomez MA Contreras I Hallé M Tremblay ML McMaster RW Olivier M. Leishmania GP63 Alters Host Signaling Through Cleavage-Activated Protein Tyrosine Phosphatases. Sci Signal (2009) 2:1–13. doi: 10.1126/scisignal.2000213 19797268

[40] Nandan D Yi T Lopez M Lai C Reiner NE. Leishmania EF-1α Activates the Src Homology 2 Domain Containing Tyrosine Phosphatase SHP-1 Leading to Macrophage Deactivation. J Biol Chem (2002) 277:50190–7. doi: 10.1074/jbc.M209210200 12384497

[41] Timm T Annoscia G Klein J Lochnit G. The Eukaryotic Elongation Factor 1 Alpha (Eef1a) From the Parasite Leishmania Infantum is Modified With the Immunomodulatory Substituent Phosphorylcholine (PC). Molecules (2017) 22:1–16. doi: 10.3390/molecules22122094 PMC6149742 29186074

[42] Barral-Netto M Barral A Brownell CE Skeiky YAW Ellingsworth LR Twardzik DR. Transforming Growth Factor-β in Leishmanial Infection: A Parasite Escape Mechanism. Science (1992) 257:545–48. doi: 10.1126/science.1636092 1636092

[43] Bhattacharya P Ghosh S Ejazi SA Rahaman M Pandey K Ravi Das VN. Induction of IL-10 and Tgfβ From CD4+CD25+Foxp3+ T Cells Correlates With Parasite Load in Indian Kala-Azar Patients Infected With Leishmania Donovani. PloS Negl Trop Dis (2016) 10:1–20. doi: 10.1371/journal.pntd.0004422 PMC4735109 26829554

[44] Mottram JC Brooks DR Coombs GH. Roles of Cysteine Proteinases of Trypanosomes and Leishmania in Host-Parasite Interactions. Curr Opin Microbiol (1998) 1:455–60. doi: 10.1016/S1369-5274(98)80065-9 10066510

[45] Somanna A Mundodi V Gedamu L. Functional Analysis of Cathepsin B-Like Cysteine Proteases From Leishmania Donovani Complex: Evidence for the Activation of Latent Transforming Growth Factor β. J Biol Chem (2002) 277:25305–12. doi: 10.1074/jbc.M203034200 12000761

[46] Gaur U Roberts SC Dalvi RP Corraliza I Ullman B Wilson ME. An Effect of Parasite-Encoded Arginase on the Outcome of Murine Cutaneous Leishmaniasis. J Immunol (2007) 179:8446–53. doi: 10.4049/jimmunol.179.12.8446 18056391

[47] Mou Z Muleme HM Liu D Jia P Okwor IB Kuriakose SM. Parasite-Derived Arginase Influences Secondary Anti- Leishmania Immunity by Regulating Programmed Cell Death-1–Mediated CD4 + T Cell Exhaustion. J Immunol (2013) 190:3380–9. doi: 10.4049/jimmunol.1202537 PMC3737427 23460745

[48] Kamir D Zierow S Leng L Cho Y Diaz Y Griffith J. A Leishmania Ortholog of Macrophage Migration Inhibitory Factor Modulates Host Macrophage Responses. J Immunol (2008) 180:8250–61. doi: 10.4049/jimmunol.180.12.8250 PMC2668862 18523291

[49] Holowka T Castilho TM Garcia AB Sun T McMahon-Pratt D Bucala R. Leishmania-Encoded Orthologs of Macrophage Migration Inhibitory Factor Regulate Host Immunity to Promote Parasite Persistence. FASEB J (2016) 30:2249–65. doi: 10.1096/fj.201500189R PMC4871794 26956417

[50] Sommerville C Richardson JM Williams RAM Mottram JC Roberts CW Alexander J. Biochemical and Immunological Characterization of Toxoplasma Gondii Macrophage Migration Inhibitory Factor. J Biol Chem (2013) 288:12733–41. doi: 10.1074/jbc.M112.419911 PMC3642319 23443656

[51] Parks DE Rodriguez M Weigle WO Ortiz-ortiz L Parks DE Rodriguez M. Polyclonal B Lymphocyte Activation During Trypanosoma Cruzi Infection. J Immunol (1980) 124:121–6. 6153088

[52] Flavia NA Morrot A Freire-de-Lima CG. Immune Evasion Strategies of Trypanosoma Cruzi. J Immunol Res (2015) 2015:178947. doi: 10.1155/2015/178947 26240832 PMC4512591

[53] Reina-San-Martín B Degrave W Rougeot C Cosson A Chamond N Cordeiro-da-Silva A. A B-Cell Mitogen From a Pathogenic Trypanosome is a Eukaryotic Proline Racemase. Nat Med (2000) 6:890–7. doi: 10.1038/78651 10932226

[54] Gao W Wortis HH Pereira MA. The Trypanosoma Cruzi Trans-Sialidase is a T Cell-Independent B Cell Mitogen and an Inducer of non-Specific Ig Secretion. Int Immunol (2002) 14:299–308. doi: 10.1093/intimm/14.3.299 11867566

[55] Bermejo DA Jackson SW Gorosito-Serran M Acosta-Rodriguez EV Amezcua Vesely MC Sather BD. Trypanosoma Cruzi Trans-Sialidase Initiates a Program Independent of the Transcription Factors Rorγt and Ahr That Leads to IL-17 Production by Activated B Cells. Nat Immunol (2013) 14:514–22. doi: 10.1038/ni.2569 PMC3631452 23563688

[56] Rodrigues AA Clemente TM dos Santos MA Machado FC Gomes RGB Moreira HHT. A Recombinant Protein Based on Trypanosoma Cruzi P21 Enhances Phagocytosis. PloS One (2012) 7:1–9. doi: 10.1371/journal.pone.0051384 PMC3519637 23251513

[57] Santos MAD Teixeira FB Moreira HHT Rodrigues AA MacHado FC Clemente TM. A Successful Strategy for the Recovering of Active P21, an Insoluble Recombinant Protein of Trypanosoma Cruzi. Sci Rep (2014) 4:1–6. doi: 10.1038/srep04259 PMC3941101 24590372

[58] Teixeira TL Machado FC Alves Da Silva A Teixeira SC Borges BC Dos Santos MA. Trypanosoma Cruzi P21: A Potential Novel Target for Chagasic Cardiomyopathy Therapy. Sci Rep (2015) 5:1–10. doi: 10.1038/srep16877 PMC4648062 26574156

[59] Nogueira N Bianco C Zanil C. Brief Definitive Report STUDIES on the SELECTIVE LYSIS and PURIFICATION of Localization, and Fate of Trypanosoma Cruzi Within Mouse Peritoneal Macro- Phages. Employing the Mixed Population of Parasites Available From Culture Medium, it had Been Noted T. J Exp Med (1975) 142:224–9. doi: 10.1084/jem.142.1.224 PMC2189871 807672

[60] Fischer E Ouaissi MA Velge P Cornette J Kazatchkine MD. Gp 58/68, a Parasite Component That Contributes to the Escape of the Trypomastigote Form of T. Cruzi From Damage by the Human Alternative Complement Pathway. Immunol (1988) 65:299–303. PMC1384928 2973433

[61] Ferreira V Valck C Sánchez G Gingras A Tzima S Molina MC. The Classical Activation Pathway of the Human Complement System is Specifically Inhibited by Calreticulin From Trypanosoma Cruzi. J Immunol (2004) 172:3042–50. doi: 10.4049/jimmunol.172.5.3042 14978109

[62] Sosoniuk E Vallejos G Kenawy H Gaboriaud C Thielens N Fujita T. Trypanosoma Cruzi Calreticulin Inhibits the Complement Lectin Pathway Activation by Direct Interaction With L-Ficolin. Mol Immunol (2014) 60:80–5. doi: 10.1016/j.molimm.2014.03.014 24769495

[63] Valck C Ramírez G López N Ribeiro CH Maldonado I Sánchez G. Molecular Mechanisms Involved in the Inactivation of the First Component of Human Complement by Trypanosoma Cruzi Calreticulin. Mol Immunol (2010) 47:1516–21. doi: 10.1016/j.molimm.2010.01.019 20153898

[64] Park SY Kim IS. Engulfment Signals and the Phagocytic Machinery for Apoptotic Cell Clearance. Exp Mol Med (2017) 49:e331–10. doi: 10.1038/emm.2017.52 PMC5454446 28496201

[65] Ramírez G Valck C Molina MC Ribeiro CH López N Sánchez G. Trypanosoma Cruzi Calreticulin: A Novel Virulence Factor That Binds Complement C1 on the Parasite Surface and Promotes Infectivity. Immunobiology (2011) 216:265–73. doi: 10.1016/j.imbio.2010.04.001 20472323

[66] Eakin AE Mills AA Harth G McKerrow JH Craik CS. The Sequence, Organization, and Expression of the Major Cysteine Protease (Cruzain) From Trypanosoma Cruzi. J Biol Chem (1992) 267:7411–20. doi: 10.1016/s0021-9258(18)42533-1 1559982

[67] Ferrão PM D’Avila-Levy CM Araujo-Jorge TC Degrave WM Gonçalves ADS Garzoni LR. Cruzipain Activates Latent TGF-β From Host Cells During T. Cruzi Invasion. PloS One (2015) 10:1–15. doi: 10.1371/journal.pone.0124832 PMC4418758 25938232

[68] Waghabi MC Keramidas M Feige JJ Araujo-Jorge TC Bailly S. Activation of Transforming Growth Factor β by Trypanosoma Cruzi. Cell Microbiol (2005) 7:511–7. doi: 10.1111/j.1462-5822.2004.00481.x 15760451

[69] Estrada D Specker G Martínez A Dias PP Hissa B Andrade LO. Cardiomyocyte Diffusible Redox Mediators Control Trypanosoma Cruzi Infection: Role of Parasite Mitochondrial Iron Superoxide Dismutase. Biochem J (2018) 475:1235–51. doi: 10.1042/BCJ20170698 29438066

[70] Paiva CN Medei E Bozza MT. ROS and Trypanosoma Cruzi: Fuel to Infection, Poison to the Heart. PloS Pathog (2018) 14:1–19. doi: 10.1371/journal.ppat.1006928 PMC5908069 29672619

[71] dos Santos GP Abukawa FM Souza-Melo N Alcântara LM Bittencourt-Cunha P Moraes CB. Cyclophilin 19 Secreted in the Host Cell Cytosol by Trypanosoma Cruzi Promotes ROS Production Required for Parasite Growth. Cell Microbiol (2021) 23:e13295. doi: 10.1111/cmi.13295 33222354 PMC7954913

[72] Bach JF. The Hygiene Hypothesis in Autoimmunity: The Role of Pathogens and Commensals. Nat Rev Immunol (2018) 18:105–20. doi: 10.1038/nri.2017.111 29034905

[73] Summers RW Elliot DE Urban JF Thompson R Weinstock JV. Trichuris Suis Therapy in Crohn’s Disease. Gut (2005) 54:87–90. doi: 10.1136/gut.2004.041749 15591509 PMC1774382

[74] Summers RW Elliott DE Urban JF Thompson RA Weinstock JV. Trichuris Suis Therapy for Active Ulcerative Colitis: A Randomized Controlled Trial. Gastroenterology (2005) 128:825–32. doi: 10.1053/j.gastro.2005.01.005 15825065

[75] Sandborn WJ Elliott DE Weinstock J Summers RW Landry-Wheeler A Silver N. Randomised Clinical Trial: The Safety and Tolerability of Trichuris Suis Ova in Patients With Crohn’s Disease. Aliment Pharmacol Ther (2013) 38:255–63. doi: 10.1111/apt.12366 23730956

[76] Schölmerich J Fellermann K Seibold FW Rogler G Langhorst J Howaldt S. A Randomised, Double-Blind, Placebo-Controlled Trial of Trichuris Suis Ova in Active Crohn’s Disease. J Crohn’s Colitis (2017) 11:390–9. doi: 10.1093/ecco-jcc/jjw184 PMC5881737 27707789

[77] Huang X Zeng L Chen F Zhu J Zhu M. Trichuris Suis Ova Therapy in Inflammatory Bowel Disease: A Meta-Analysis. Med (2018) 97:e12087. doi: 10.1097/MD.0000000000012087 PMC6113037 30142867

[78] Bager P Arnved J Rønborg S Wohlfahrt J Poulsen LK Westergaard T. Trichuris Suis Ova Therapy for Allergic Rhinitis: A Randomized, Double-Blind, Placebo-Controlled Clinical Trial. J Allergy Clin Immunol (2010) 125:123–30. doi: 10.1016/j.jaci.2009.08.006 19800680

[79] Bisht N Khatri V Chauhan N Kalyanasundaram R. Cystatin From Filarial Parasites Suppress the Clinical Symptoms and Pathology of Experimentally Induced Colitis in Mice by Inducing T-Regulatory Cells, B1-Cells, and Alternatively Activated Macrophages. Biomedicines (2019) 7:85. doi: 10.3390/biomedicines7040085 PMC6966632 31683524

[80] Jang SW Cho MK Park MK Kang SA Na BK Ahn SC. Parasitic Helminth Cystatin Inhibits DSS-Induced Intestinal Inflammation *via* IL-10 +F4 + Macrophage Recruitment. Korean J Parasitol (2011) 49:245–54. doi: 10.3347/kjp.2011.49.3.245 PMC3210841 22072824

[81] Wang S Xie Y Yang X Wang X Yan K Zhong Z. Therapeutic Potential of Recombinant Cystatin From Schistosoma Japonicum in TNBS-Induced Experimental Colitis of Mice. Parasites Vectors (2016) 9:1–13. doi: 10.1186/s13071-015-1288-1 26728323 PMC4700642

[82] Smyth DJ Ren B White MPJ McManus C Webster H Shek V. Oral Delivery of a Functional Algal-Expressed TGF-β Mimic Halts Colitis in a Murine DSS Model. J Biotechnol (2021) 340:1–12. doi: 10.1016/j.jbiotec.2021.08.006 34390759 PMC8516079

[83] Saeij JP Boyle JP Coller S Taylor S Sibley LD Brooke-Powell ET. Polymorphic Secreted Kinases are Key Virulence Factors in Toxoplasmosis. Science (2006) 314:1780–3. doi: 10.1126/science.1133690 PMC2646183 17170306

[84] Blader IJ Saeij JP. Communication Between Toxoplasma Gondii and its Host: Impact on Parasite Growth, Development, Immune Evasion, and Virulence. Apmis (2009) 117:458–76. doi: 10.1111/j.1600-0463.2009.02453.x PMC2810527 19400868

[85] Melo MB Jensen KDC Saeij JPJ. Toxoplasma Gondii Effectors are Master Regulators of the Inflammatory Response. Trends Parasitol (2011) 27:487–95. doi: 10.1016/j.pt.2011.08.001 PMC3200456 21893432

[86] Xu YW Xing RX Zhang WH Li L Wu Y Hu J. Toxoplasma ROP16I/III Ameliorated Inflammatory Bowel Diseases *via* Inducing M2 Phenotype of Macrophages. World J Gastroenterol (2019) 25:6634–52. doi: 10.3748/wjg.v25.i45.6634 PMC6906210 31832003

[87] Xie Y Wen H Yan K Wang S Wang X Chen J. Toxoplasma Gondii GRA15 II Effector-Induced M1 Cells Ameliorate Liver Fibrosis in Mice Infected With Schistosomiasis Japonica. Cell Mol Immunol (2018) 15:120–34. doi: 10.1038/cmi.2016.21 PMC5811674 27157496

[88] Lapara NJ Kelly BL. Suppression of LPS-Induced Inflammatory Responses in Macrophages Infected With Leishmania. J Inflammation (2010) 7:1–9. doi: 10.1186/1476-9255-7-8 PMC2824668 20205812

[89] Chandra D Naik S. Leishmania Donovani Infection Down-Regulates TLR2-Stimulated IL-12p40 and Activates IL-10 in Cells of Macrophage/Monocytic Lineage by Modulating MAPK Pathways Through a Contact-Dependent Mechanism. Clin Exp Immunol (2008) 154:224–34. doi: 10.1111/j.1365-2249.2008.03741.x PMC2612710 18778366

[90] Cheekatla SS Aggarwal A Naik S. Mtor Signaling Pathway Regulates the IL-12/IL-10 Axis in Leishmania Donovani Infection. Med Microbiol Immunol (2012) 201:37–46. doi: 10.1007/s00430-011-0202-5 21567173

[91] Lundberg AM Hansson GK. Innate Immune Signals in Atherosclerosis. Clin Immunol (2010) 134:5–24. doi: 10.1016/j.clim.2009.07.016 19740706

[92] Brand S. Crohn’s Disease: Th1, Th17 or Both? The Change of a Paradigm: New Immunological and Genetic Insights Implicate Th17 Cells in the Pathogenesis of Crohn’s Disease. Gut (2009) 58:1152–67. doi: 10.1136/gut.2008.163667 19592695

[93] Finckh A. Early Inflammatory Arthritis Versus Rheumatoid Arthritis. Curr Opin Rheumatol (2009) 21:118–23. doi: 10.1097/BOR.0b013e3283235ac4 19339921

[94] Saresella M Basilico N Marventano I Perego F La Rosa F Piancone F. Leishmania Infantum Infection Reduces the Amyloid β42-Stimulated NLRP3 Inflammasome Activation. Brain Behav Immun (2020) 88:597–605. doi: 10.1016/j.bbi.2020.04.058 32335194

[95] Heneka MT Kummer MP Stutz A Delekate A Schwartz S Vieira-Saecker A. NLRP3 is Activated in Alzheimer’s Disease and Contributes to Pathology in APP/PS1 Mice. Nature (2013) 493:674–8. doi: 10.1038/nature11729 PMC3812809 23254930

[96] Higuchi MDL. Trypanosoma Cruzi Trans-Sialidase as a New Therapeutic Tool in the Treatment of Chronic Inflammatory Diseases: Possible Action Against Mycoplasma and Chlamydia. Med Hypotheses (2004) 63:616–23. doi: 10.1016/j.mehy.2004.03.043 15325005

[97] Tang MLK Mullins RJ. Food Allergy: Is Prevalence Increasing? Intern Med J (2017) 47:256–61. doi: 10.1111/imj.13362 28260260

[98] Asher MI Montefort S Björkstén B Lai CK Strachan DP Weiland SK. Worldwide Time Trends in the Prevalence of Symptoms of Asthma, Allergic Rhinoconjunctivitis, and Eczema in Childhood: ISAAC Phases One and Three Repeat Multicountry Cross-Sectional Surveys. Lancet (2006) 368:733–43. doi: 10.1016/S0140-6736(06)69283-0 16935684

[99] Safiri S Kolahi AA Hoy D Smith E Bettampadi D Mansournia MA. Global, Regional and National Burden of Rheumatoid Arthritis 1990-2017: A Systematic Analysis of the Global Burden of Disease Study 2017. Ann Rheum Dis (2019) 78:1463–71. doi: 10.1136/annrheumdis-2019-215920 31511227

[100] Molodecky NA Soon IS Rabi DM Ghali WA Ferris M Chernoff G. Increasing Incidence and Prevalence of the Inflammatory Bowel Diseases With Time, Based on Systematic Review. Gastroenterology (2012) 142:46–54.e42. doi: 10.1053/j.gastro.2011.10.001 22001864

[101] Shivashankar R Tremaine WJ Harmsen WS Loftus EV. Incidence and Prevalence of Crohn’s Disease and Ulcerative Colitis in Olmsted County, Minnesota From 1970 Through 2010. Clin Gastroenterol Hepatol (2017) 15:857–63. doi: 10.1016/j.cgh.2016.10.039 PMC5429988 27856364

[102] Shimokawa C Kato T Takeuchi T Ohshima N Furuki T Ohtsu Y. CD8+ Regulatory T Cells are Critical in Prevention of Autoimmune-Mediated Diabetes. Nat Commun (2020) 11:1992. doi: 10.1038/s41467-020-15857-x 32321922 PMC7176710

